# Multiple introns in a deep-sea Annelid (*Decemunciger*: Ampharetidae) mitochondrial genome

**DOI:** 10.1038/s41598-017-04094-w

**Published:** 2017-06-27

**Authors:** Angelo F. Bernardino, Yuanning Li, Craig R. Smith, Kenneth M. Halanych

**Affiliations:** 1 Universidade Federal do Espírito Santo, Grupo de Ecologia Bêntica, Departamento de Oceanografia, Av. Fernando Ferrari, 514, Vitória, ES 29075-910 Brazil; 2 Auburn University, Department of Biological Sciences, 101 Life Sciences Building, Auburn, AL 36849 USA; 3 Department of Oceanography, SOEST, University of Hawaii at Manoa, 1000 Pope Road, Honolulu, HI 96822 USA

## Abstract

Wood falls provide episodic fluxes of energy to the sea floor that are degraded by a species-rich benthic fauna. Part of this rich diversity includes annelid polychaetes but unfortunately, our understanding of such fauna is limited and their genetic variability and evolutionary origins remain poorly known. In this study, we sequenced complete mitochondrial genomes from three congeneric *Decemunciger* (Ampharetidae) individuals that had colonized multiple wood falls in the deep (~1600 m) NE Pacific Ocean. Mitochondrial gene order within *Decemunciger* was similar to the three other available Terebellomorpha genomes, consistent with the relatively conserved nature of mitochondrial genomes within annelids. Unexpectedly, we found introns within the *cox1*, *nad1* and *nad4* genes of all three genomes assembled. This is the greatest number of introns observed in annelid mtDNA genomes, and possibly in bilaterians. Interestingly, the introns were of variable sizes suggesting possible evolutionary differences in the age and origins of introns. The sequence of the introns within *cox1* is similar to Group II introns previously identified, suggesting that introns in the mitochondrial genome of annelids may be more widespread then realized. Phylogenetically, *Decemunciger* appears to be a sister clade among current vent and seep deep-sea Ampharetinae.

## Introduction

Ampharetid polychaetes are tube-dewelling annelids that are abundant on shallow-marine and deep-sea continental margins, with some species showing adaptations to sulfide-rich sediments near cold seeps and organic falls, including wood-falls and whale carcasses^1–7^. In organic-fall and cold-seep habitats, these polychaetes can show remarkable abundances and diversity and may be important for organic-matter degradation^8, 9^. However, as with many other deep-sea taxa, there is limited understanding of their diversity and evolution, requiring additional study including use of informative molecular markers^10, 11^. Despite their high diversity and abundance in the deep-sea, a limited number of polychaete taxa have been molecularly characterized from deep-sea ecosystems and from chemosynthetic habitats^12–14^.

Advances in phylogenetic and evolutionary understanding of Annelida has been made using comparative mitogenomics^15–17^. Annelids, like other bilaterians, typically have 37 mitochondrial genes^18–20^. Recent descriptions of mitochondrial genomes from several annelid linneages revealed marked differences in gene order that are helping to resolve phylogenetic relationships, even though some inconsistencies between sequence data and phylogenies remain^14, 20, 21^. There are currently about 90 complete annelid mitochondrial DNA sequences (mtDNA) published^14, 22^, with many underrepresented linneages, making broad scale mitogenomic comparisons limited given the extremely high number of species in the deep-sea^13^. For instance, in the family Ampharetidae, there are only two incomplete mitochondrial genomes reported (*Eclysippe vanelli* and *Auchenoplax crinita*)^18^.

Descriptions of new mtDNA genomes can help to clarify phylogenetic relationships among closely related lineages and also to discover less frequent genome features such as the presence of group II introns^23^. The phylogeny of Terebelliformia includes two clades, one with Ampharetidae, Alvinellidae and Pectinariidae and the other with Terebellidae and Trichobranchidae^24^. Ampharetidae is a sister group to Alvinellidae based on current molecular analysis from mitochondrial and nuclear genes^6, 7, 11, 25^, but the taxonomy within the family is complex due to morphological variability. There is only limited phylogenetic work within Ampharetidae, but the subfamily Ampharetinae host several species adapted to chemosynthetic deep-sea ecosystems^7, 25^.

Group II introns are self-splicing mobile genetic elements typically found in mitochondrial and other organelle genomes in lower eukaryotes, microbes, algae and higher plants, and are reported to contain genes with mobile capability^26–28^. Within Bilateria metazoans, group II introns were first described in the mitochondrial genome of the polychaete *Nephtys* sp.^23^, even though bilaterian mtDNA genomes were thought to be conserved in terms of gene content and lack introns^17, 29^. However, recent mitogenomic investigations have revealed a more common presence of Group II introns in the *cox1* mitochondrial gene in some Annelid worms, including two *Glycera* species and one myzostomid Endomyzostoma^30, 31^. Based on previous phylogenetic analysis, Richter *et al*.^30^ demonstrated a close phylogenetic relationship between *Nephtys* sp. and *Glycera* introns, but less similarity with one of the two *cox1* introns from *Glycera fallax*. The presence of introns in a few distantly-related annelid taxa makes mechanisms of intron acquisition and substitution rates of the relevant mtDNA regions unclear^23^. Although mitochondrial gene order is relatively conserved among annelids^19^, the presence or absence of such introns, their number and their association with unique or multiple genes with variable function suggests that annelid mitochondrial genomes may exhibit more varibility than anticipated^19, 20^.

We sequenced mitochondrial genomes of an abundant ampharetid (*Decemunciger* sp.) sampled from wood-fall blocks experimentally implanted for 12 months at ~1600 m depth on the East Pacific US margin. We detected differences in mitochondrial gene order relative to previously reported Terebellomorpha mt genomes^18^. Unexpectedly, we detected three intragenetic regions within *cox1* (Group II intron), *nad1* and *nad4* genes. Furthermore, we conducted a phylogenetic analysis of Ampharetidae based on available mt genomes and transcriptomic data to further explore ampharetid evolutionary history.

## Results and Discussion

### Genome assemblies and description

**Figure 1:**
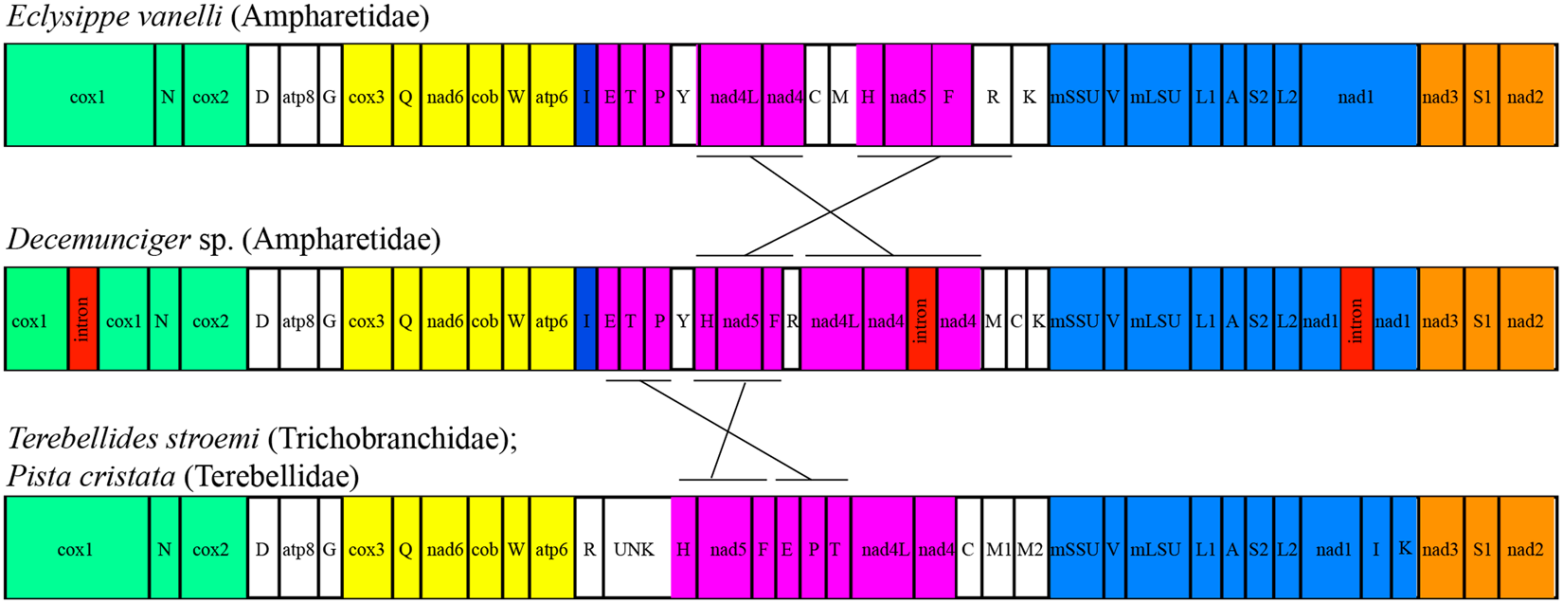
Mitochondrial gene order of *Decemunciger* sp. sequenced in this study. Conserved gene clusters are represented in different colors as in Jennings and Halanych (2005) and Zhong *et al*.^18^. Lines between genomes highlight regions with different gene order. Red box indicates the introns detected within *Decemunciger* sp. mtDNA.

Using Illumina sequence data from three individuals of a deep-sea ampharetid annelid abundant on wood-falls in the deep NE Pacific, we assembled complete mitochondrial genomes. The three individuals were morphologically identified to potentialy new species of *Decemunciger*, and all three assembled genomes had a 100% identical *cox1* gene. There is no previous molecular data to confirm the identity of *Decemunciger* sampled in wood blocks separated by over 400 km on the Oregon-Washington margin, with the paratype described from the Atlantic^32^. Using a BLAST-based approach^33^, we identified mtDNA contigs that were roughly 15,000–16,000 bp in size from the genome assembly. The integrity of these contigs was confirmed by mapping sequence reads to the assembly ^34^. *Decemunciger* sp. mt genome has 16,703–16,974 bp without the introns, which is similar to the ampharetids *Eclysippe vanelli* (16,547; EU239687^18^) and is slightly longer than the other ampharetid *Auchenoplax crinita* (13,759 bp; FJ976041 incomplete) and the Terebellomorpha *Pista cristata* (15,894 bp; EU239688). The complete mtDNA of *Decemunciger* sp. is approximately 19 kb long (19,003 to 19,274 bp; Table 1), with 2,300 bp of introns (Fig. 1; Table 1). Other previously studied annelids have mtDNA sizes between 14,414 and 22,058 bp^14, 19, 20, 22, 30^. Although the mitochondrial genome size varied slightly among our three specimens, the intergenic region between *nad2 and cox1* showed the greatest variation.

**Table 1:** Genome size, coverage, coverage depth and base composition of assembled *Decemunciger* sp.

	*Decemunciger* sp. A3359 AC KY742027	*Decemunciger* sp. A3372-1 AC KY774370	*Decemunciger* sp. A3372-2 AC KY774371
mtDNA size (bp)	19,274	19,096	19,003
Coverage depth	258x	1514x	1394x
Base composition			
Whole genome			
A	32.7%	32.7%	32.7%
T	34.4%	34.4%	34.5%
G	13.8%	13.8%	13.8%
C	19.1%	19.1%	19.1%
GC	32.9%	32.9%	32.9%
CDS			
A	29.0%	29.0%	28.9%
T	36.2%	36.3%	36.3%
G	14.5%	14.4%	14.6%
C	20.3%	20.4%	20.3%
GC	34.8%	34.8%	34.9%

Mitochondrial genomes. AC - GenBank accession numbers.

For each mitochondrial genome sequenced herein, the genome was composed of 37 genes, with all 13 protein-coding, 2 ribosomal rRNAs and 22 tRNAs^29^ (Fig. 1). All genes encoded on the same strand, typical of other annelids^20^. As observed in other Terebellomorpha, *Decemunciger* sp. mtDNA is AT rich (65.1% AT) in the coding regions (CDS) (Table 1). Mitochondrial gene orders of *Decemunciger* sp. mtDNA differ from *E. vanelli* in relation to positions of *nad4*, *nad4L* and *nad5* genes, and differs from *Terebellides stroemi* (Trichobranchidae) and *Pista cristata* (Terebellidae) in the positions of tRNAs (Fig. 1 ^18, 20^). The difference in protein coding gene order between the ampharetids *Decemunciger* sp. and *E. vanelli* support a higher varibility in gene order within Ampharetidae^19, 20^. A recent analysis of Syllidae also showed marked variability on the order of protein enconding genes, with four distinct gene orders^14^. With only 89 complete mtDNAs sequenced from annelids^14, 15, 19, 20, 22^, more variation in gene orders will certainly be uncovered. Slight differences in the number of tRNAs were also revealed in *Decemunciger* sp., if compared to previous Terebelliformia mtDNA. *Terebellides stroemi* and *P. cristata* have two copies of the methionine tRNA gene in their mtDNA, whereas only one copy was present in *Decemunciger* sp. mtDNA, as previously observed on the ampharetid *E. vanelli* ^18^. Changes in the postion of tRNAs between *Decemunciger* sp. and the other Terebellomorpha were also observed (Fig. 1), and are common in bilaterian mtDNAs^29^.

### *Introns in* Decemunciger *mtDNA*

Mitochondrial genomes of the three *Decemunciger* sp. individuals revealed the presence of introns within the *cox1*, *nad1* and *nad4* genes, which is the first report to date of multiple introns in distinct mitochondrial genes from Bilaterians. Introns within the *cox1*, *nad1* and *nad4* genes were 1648, 390 and 262 bp long, respectively. All introns were the same size across the three assembled genomes and none of these introns coded a protein, but presented palindromic sequences at both ends (based on a blast search results). The *cox1* intron contained a 390 bp ORF for an intron maturase 2 type II transcriptase (blastp e-value 7.68e-08), which was similar to other Group II introns reported in annelids^23, 30^. Although ORFs were not found in introns from *nad1* and *nad4* genes, these regions could possibly be derived form ancient transposible elements which have since lost any function. However, the intron maturase enzyme in the *cox1* intron may assist transposition of these elements^35^. Another possibility is that the *nad1* and *nad4* introns are discontinuous parts of one transposible element split among those genes and can be trans-spliced to form a functional intron^27, 36^. These mechanisms have been observed in higher plants; if true here, would be the first known case of trans-complementation of introns in annelid mitochondrial genes.

**Figure 2:**
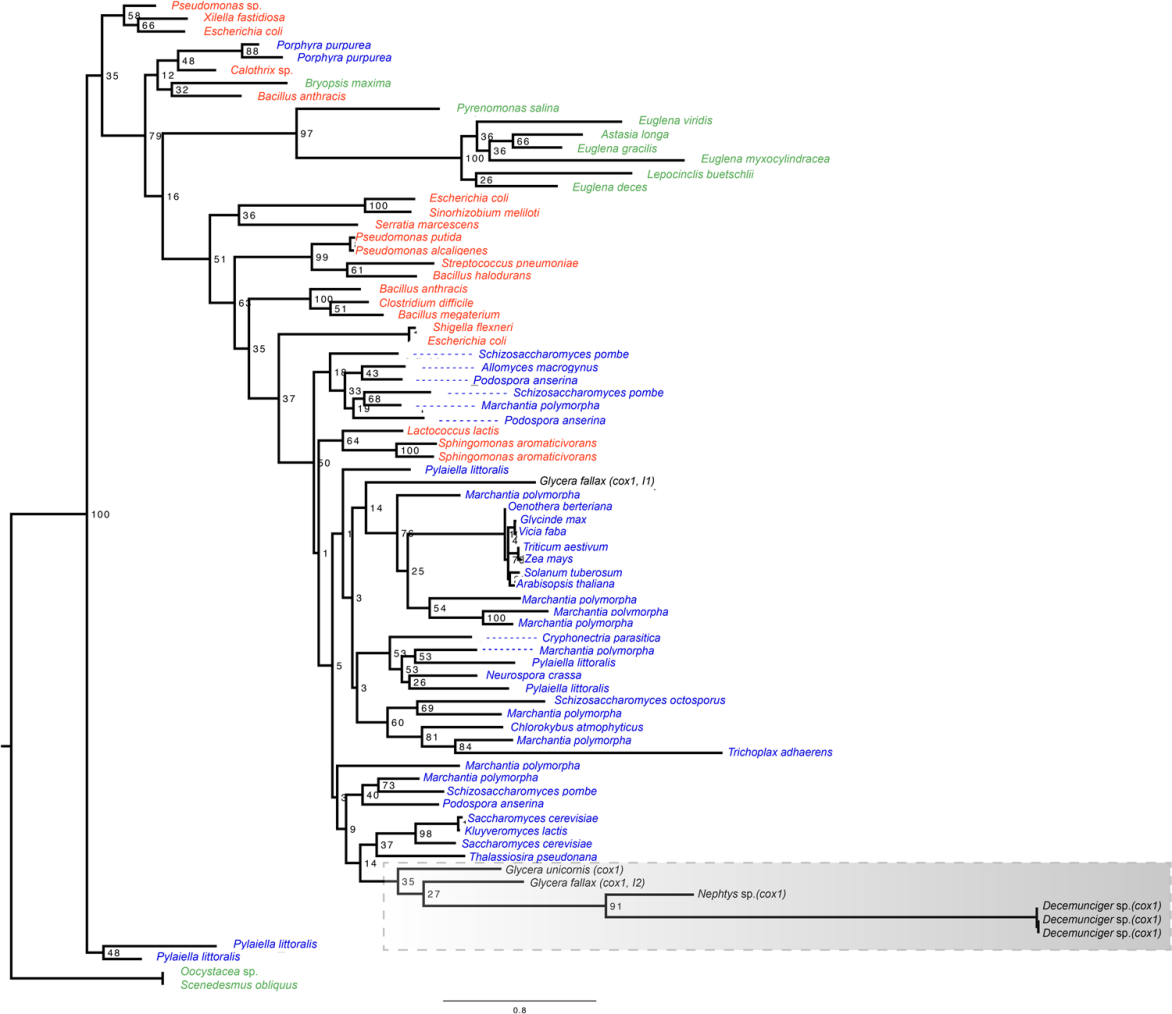
Phylogenetic position of Annelid group II introns (black colour) including *Decemunciger* sp. *cox1* intron ORF and previous tree by Richter *et al*.^30^, Valles *et al*.^23^ and Zimmerly *et al*.^27^. Outlined are host species. Color-codes as in Richter *et al*.^30^: Green – chloroplast group II intron-encoded ORFs; Blue – Mitochondrial group II intron-encoded ORFs and RED – Bacterial group II intron-encoded ORFs. Genbank numbers are given in Richter *et al*.^30^.

The insertion position into the *cox1* gene and size of the introns were identical within the three *Decemunciger* mitochondrial genomes sequenced. Multiple introns were first identified on mitochondrial genes (*cox1* and *nad5*) of sea anemones (Group I intron^37^, and recently Group II introns have been reported on a *cox1* gene of a Nephtydae (*Nephtys* sp.) and glycerid polychaetes^23, 30^. Intron sizes, their position within the *cox1* gene and their coding protein sequences, differ between *Nephtys* sp., *Decemunciger* sp. and *Glycera* spp., consistent with distinct episodes of intron gain in these annelid lineages^23, 38^. Phylogenetic differences in the ORF region between introns are evident (Fig. 2). Different insertion positions of introns within *cox1* genes of *Decemunciger* sp., *Nephtys* sp. and *Glycera* spp. may be a result of variable intronic target sites (IEP) within the mitochondrial genome (Fig. 2)^27, 30^. The *cox1* intron in *Nephtys* sp. has 1819 bp, whereas it is slightly shorter (1647 bp) in *Decemunciger* sp. The *Nephtys* sp. intron has an ORF region of 525 bp coding a reverse transcriptase enzime, whereas the 390 bp region within the *Decemunciger* sp. *cox1* gene translates into a type II intron maturase enzyme. Amino acid sequences of both *Nephtys* sp. and *Decemunciger* sp. introns are also only 16% similar, further supporting independent events of insertion in a scenario of “late intron-gain” for annelids^23, 38^.

*Nephtys* sp. and *Decemunciger* sp. represent distinct linneages among Annelida, which likely inherited introns from separate viral vectors. The limited presence of introns may also suggest a high rate of intron loss among lineages. The loss of introns in genomes is generally related to fast replication rates observed, for example, in microbes in a process known as “genome streamlining”^27^. Since mitochondrial DNA is considered to possess a fast evolutionary rate^39^, introns may be rapidly removed from mitochondrial genes. Further complete mtDNA sequencing will very likely reveal new patterns of introns as usual mitochondrial barcoding (e.g. *cox1*) in marine invertebrates are based on short (about 600 bp) sequences that would not detect these introns.

### Ampharetid phylogeny based on mtDNA

**Figure 3:**
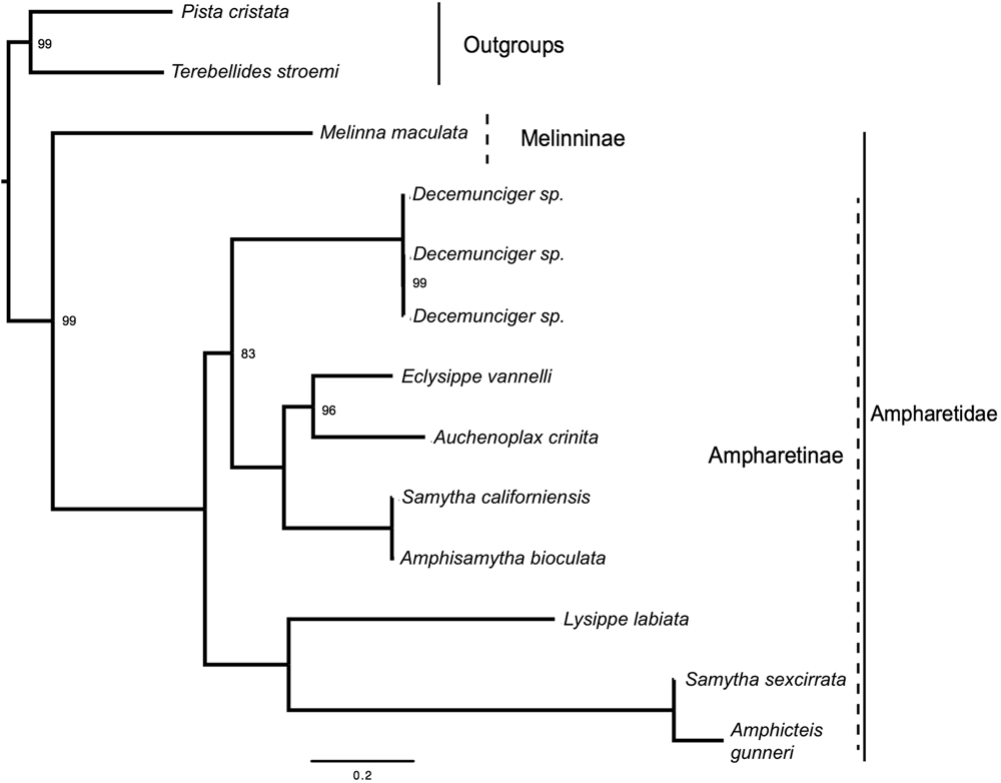
Maximum likelihood tree obtained when analyzing amino acid sequences from mtDNA protein coding genes. All nodes were supported with 100% bootstrap value (bs = 100) unless otherwise noted. Dashed lines indicate subfamilies represented within Ampharetidae.

Amino acid (AA) sequences of protein coding genes from the three mitochondrial genomes from this study, from published genomes in GenBank and from transcriptomic data (see Table 2) were used to reconstruct a phylogenetic relationship of *Decemunciger* sp. within Ampharetidae. Phylogenetic relationships of ampharetids were infered using maximum likelihood (ML) analysis from a dataset with the 10 protein-coding and 2 rRNA mitochondrial genes (see methods). The dataset contained 3,024 amino acid residues after trimming using Gblocks and the resulting ML analysis yielded a tree topology with relatively high bootstrap support values for the division of Ampharetidae subfamilies Melinninae and Ampharetinae^11, 18^ (Fig. 3). Ampharetidae was recovered as a monophyletic group, but our analysis did not include Alvinellidae^7, 25^. Melinninae and Ampharetinae were recovered as sister taxa, which supports current phylogenetic analysis^25^. Ampharetinae was also recovered as a monophyletic clade with strong support in the amino acid dataset, consistent with previous molecular and morphological analyses^7, 11, 18, 25^. Whithin Ampharetinae, the *Decemunciger* lineage was sister to a strongly supported clade (bs = 100) comprised of *Eclysippe*, *Auchenoplax*, *Samytha* and *Amphisamytha* species (Fig. 3, Supplemental Fig. S1). *Decemunciger* has also marked morphological similarities (e.g. branchiae position and number) with the vent ampharetid genus *Paramytha* gen nov., which is a sister group to other vent/seep Ampharetinae clades based on *cox1*, *16S* and *18S* genes^25, 32^. In summary, our phylogenetic analysis support *Decemunciger* as within the Ampharetinae, within a clade comprised of several described species from chemosynthetic ecosystems in the North Atlantic and Arctic basins.

## Methods

### Genome assembly, annotation and mapping

Three Ampharetid specimens (A3359, A3372–1 and A3372–2) were collected from 1.5 kg blocks of douglas fir (*Pseudotsuga menziesi)* experimentally deployed on the seafloor for 15 months and recovered via accoustic release using the *R/V Oceanus*. Ampharetid A3359 was sampled from one wood block recovered from 1605 m depth on Jun 22^nd^ 2014 (43°54.22 N; 125°10.238 W), whereas ampharetids A3372–1 and A3372–2 were sampled from wood blocks recovered about 400 km north from the previous site at 1596 m depth on Jun 27^th^ 2014 (47°57.462 N; 126o02.118 W). Morphological observations indicate that all the three specimens belonged to the ampharetid genus *Decemunciger* sp. Specimens were immediately preserved onboard in 95–100% ethanol and later transferred to Auburn University.

DNA was extracted using the DNeasy Blood & Tissue Kit (Qiagen) following manufacture’s protocols. Sequencing of genomic DNA was performed by The Genomic Services Lab at the Hudson Alpha Institute in Huntsville, Alabama on an Illumina HiSeq 2500 platform (San Diego, California) using 2 × 150 paired-end v4 chemistry. Paired-end reads were assembled *de novo* using Ray 2.2.0 with k-mer = 31^34^. Contigs of interest where identified by using blast with previously published terebellomorph mtDNA genomes^18^ against the assembled genomic data. Annotation of the 13 protein-coding genes, 2 ribosomal RNAs and tRNAs was conducted initially with MITOS web server^40^, followed by manual genome annotation in Artemis^41^. Start and stop positions of genes were confirmed by BLASTn and BLASTp^33^ searches against the partial mitochondrial genome from *Eclysippe vanelli* (GenBank Accession EU239687) as well as manual inspection.

The presence of introns within coding mitochondrial genes was confirmed by mapping the paired Illumina reads against the assembled mitochondrial genome to check for coverage in each coding region and near the intronic reads^34^ (Supplementary Fig. S2). Reads were mapped with Bowtie2^42^, indexed and sorted with Samtools and visually checked with Tablet software^43^. Identity on introns was aided by Blast searches when possible.

### Transcriptomic data generation and assembly for phylogenetic analysis

Upon collection, all specimens were either stored at −80 °C, in ethanol or preserved in RNAlater (Life Technologies Inc.). Due to a limiting amount of tissue, only RNA was extracted since mitochondrial protein-coding and ribosomal RNA genes, which were used in mitogenomic analysis, can be recovered from transcriptome sequencing^34, 44^. RNA extraction and cDNA preparation for high-throughput sequencing followed^45^. Briefly, total RNA was extracted using TRIzol (Invitrogen) and purified using the RNeasy kit (Qiagen) with on-column DNase digestion. Next, single strand cDNA libraries were reverse transcribed using the SMART cDNA Library Construction kit (Clontech) followed by double-stranded cDNA synthesis using the Advantage 2 PCR system (Clontech). Illumina sequencing library preparation and sequencing of *Lysippe labiata, Samytha sexcirrata, Samytha californiensis, Amphisamytha bioculata, Amphicteis gunneri, Auchenoplax crinita* and *Melinna maculata* were performed by The Genomic Services Lab at the Hudson Alpha Institute in Huntsville, Alabama using 2 × 100 paired-end sequencing on an Illumina HiSeq 2000 platform (San Diego, California).

Prior to assembly, Illumina paired-end transcriptome sequence data were digitally normalized to a k-mer coverage of 30 using *normalize-by-median.py* ^46^. Remaining reads were then assembled using Trinity r2013-02-25 with default settings^47^. Mitochondrial protein-coding genes and ribosomal RNAs were identified by TBLSTX and BLASTN^33^, respectively (using the recovered *E. vanelli* mt genome as query).

### Phylogenetic analysis

Fourteen taxa were included in the phylogenetic analysis. *Pista cristata* (Terebellidae) and *Terebellides stroemi* (Trichobranchidae) were acquired from GenBank (Table 2) and selected as outgroups based on data availability as well as current understanding of annelid evolutionary history^15, 20^. To assist in phylogenetic analysis and check the previous incomplete assembly of the ampharetid mtDNA *Eclysippe vanelli* ^18^, we assembled a new complete mitochondrial genome from the ampharetid *E. vanelli*. The assembled *E. vanelli* genome has an identical gene order with the previous incomplete genome and a *cox1* amino acid identity of 99.8% with the *cox1* gene from the incomplete *E. vanelli* genome^18^. We used the complete *E. vanelli* genes for phylogenetic analysis (indicated below), and included genes from transcriptomic assembly from seven other species of interest.

**Table 2:** List of taxa included in the Ampharetidae phylogenetic analysis, with genbank assession numbers and references to published sequences.

Species	Family	Subfamily	mtDNA genome	Transcriptome data	Ref
*Pista cristata*	Terebellidae		NC_011011.1		Zhong *et al*.^18^
*Terebellides stroemi*	Trichobranchidae		NC_011014		Zhong *et al*.^18^
*Decemunciger* sp A3359	Ampharetidae	Ampharetinae	this study KY742027		
*Decemunciger* sp A3372-1	Ampharetidae	Ampharetinae	this study KY774370		
*Decemunciger* sp A3372-	Ampharetidae	Ampharetinae	this study KY774371		
*Amphisamytha bioculata*	Ampharetidae	Ampharetinae		this study KY972369-KY972532	
*Samytha californiensis*	Ampharetidae	Ampharetinae		this study KY972369-KY972532	
*Samytha sexcirrata*	Ampharetidae	Ampharetinae		this study KY972369-KY972532	
*Melinna maculata*	Ampharetidae	Melinninae		this study KY972369-KY972532	
*Auchenoplax crinita*	Ampharetidae	Ampharetinae	FJ976041.1	this study KY972369-KY972532	Zhong *et al*.^18^
*Eclysippe vanelli*	Ampharetidae	Ampharetinae	this study		
*Eclysippe vanelli*	Ampharetidae	Ampharetinae	EU239687		Zhong *et al*.^18^
*Amphicteis gunneri*	Ampharetidae	Ampharetinae		this study KY972369-KY972532	
*Lysippe labiata*	Ampharetidae	Ampharetinae		this study KY972369-KY972532	

Our data set was based on amino acid sequences from 10 mitochondrial protein-coding genes (*cox1*, *cox2*, *cox3*, *cob*, *atp6*, *nad1*, *nad2*, *nad4*, *nad5*, *nad6*) and two ribosomal RNA genes (*rrnS* and *rrnL*). *nad4l*, *atp8* and *nad3* sequences were excluded due to limited number of recovered sequences from transcriptome data. Each of the 12 genes was individually aligned using MAFFT^48^ followed by manual correction. The selected genes were then trimmed using the defalut setting in Gblocks^49^ to remove ambiguously aligned regions. Genes were then concatenated into final supermatrix datasets using FASconCAT^50^ for downstream phylogenetic analysis. Phylogenetic relationships of ampharetids were infered using maximum likelihood (ML) in RAxML^51^. Prior to ML analyses, PartitionFinderV1.1.1^52^ was used to evaluate best-fit partition schemes and associated best-fit substitution models for both datasets. Topological robustness for the ML analysis was evaluated with 100 replicates of fast-bootstrapping.

### Intron phylogeny

Phylogenetic position of group II introns was compared with the alignment of which built upon an analysis by Richter *et al*.^30, 53, 54^. The mitochondrial group II introns from *cox1* genes of the Annelids *Glycera fallax*, *Glycera unicornis* and *Nephtys* sp. were analyzed and compared to the *cox1* intron ORF from *Decemunciger* sp. and other chroloplast and bacterial intronic ORFs. The Maximum likelihood analysis was conducted with RAxML v.8.0.5 under the substitution model LG + I + G + F. Bootstrap support values (>50%) from 1,000 pseudoreplicates are given at the nodes. Colorcodes were defined accordingly to Richter *et al*.^30^, where group II intron-encoded ORFs known from chloroplast genomes are highlighted in green, mitochondrial genomes in blue, and bacterial genomes in red. GenBank numbers from intron sequences used in this analysis are given in Richter *et al*.^30^.

## Electronic supplementary material

Supplementary material

Dataset 1
