# Supplementary material for: Multiple introns in a deep-sea Annelid (Decemunciger: Ampharetidae) mitochondrial genome
Source: Sci Rep. 2017 Jun 27;7:4295. doi: 10.1038/s41598-017-04094-w (PMC5487361; doi:10.1038/s41598-017-04094-w)
Supplement: Supplementary file 1 — Supplementary material [file 41598_2017_4094_MOESM1_ESM.pdf]

Supplemental material

Multiple introns in a deep-sea Annelid (*Decemunciger*: Ampharetidae) mitochondrial genome

Angelo F. Bernardino, Yuanning Li, Craig R. Smith, Kenneth M. Halanych

Figure S1

Maximum likelihood tree obtained when analyzing nucleotide sequences from mtDNA protein coding genes. All nodes were supported with 100% bootstrap value (bs=100) unless otherwise noted.

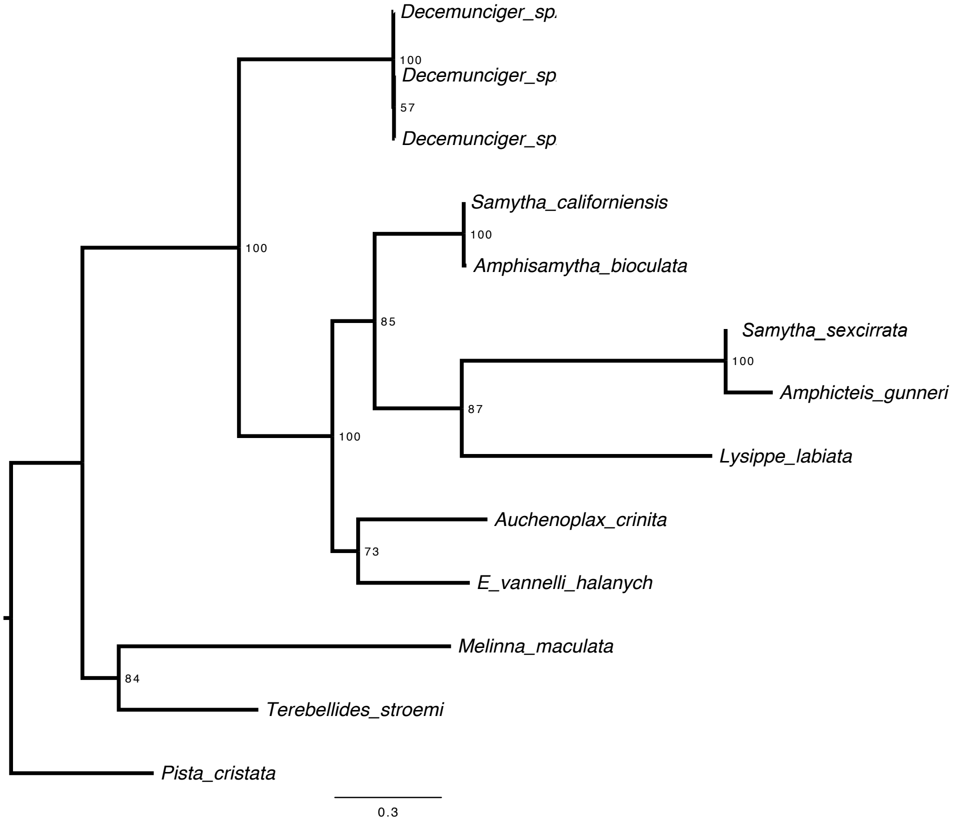

Figure S2

Mapping results for Decemunciger sp. mtDNA, indicating the coverage along the intron positions (nad1, cox1 and nad4). Introns are illustrated by dark bars.

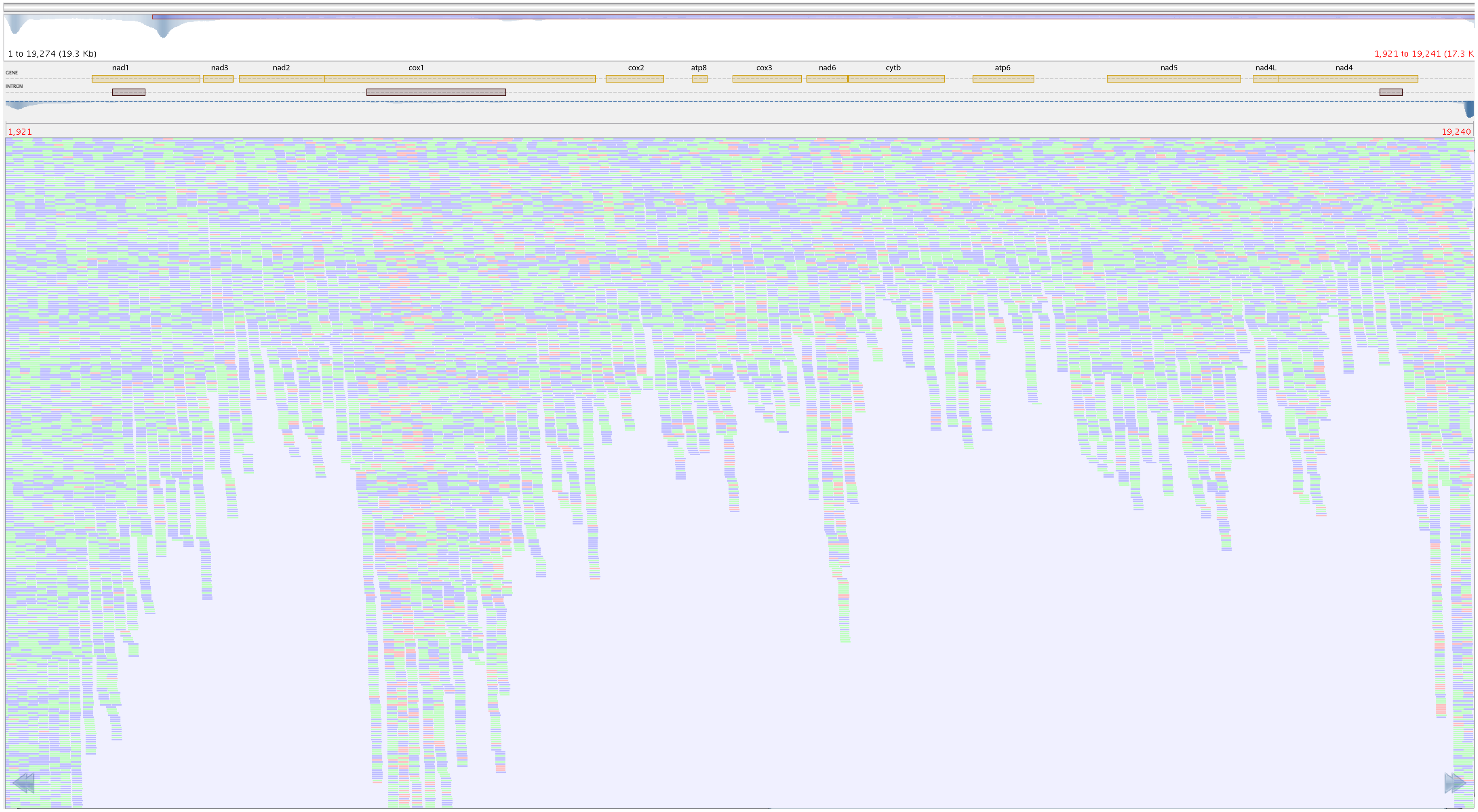

1 to 19,274 (19.3 Kb)
1,921 to 19,241 (17.3 K
GENE
INTRON
nad1
nad3
nad2
cox1
cox2
atp8
cox3
nad6
cytb
atp6
nad5
nad4L
nad4
1,921
19,240
